# Cateterismo de senos petrosos inferiores en el diagnóstico del síndrome de Cushing ACTH-dependiente: experiencia en un hospital terciario

**DOI:** 10.1515/almed-2022-0039

**Published:** 2022-10-03

**Authors:** Isabel Moreno Parro, David Ortiz Sánchez, Rosa García Moreno, Rubén Gómez Rioja, Remedios Frutos Martínez, Cristina Álvarez-Escolá

**Affiliations:** Servicio de Análisis Clínicos, Hospital Universitario La Paz – Carlos III – Cantoblanco, Madrid, España; Servicio de Endocrinología y Nutrición, Hospital Universitario La Paz – Carlos III – Cantoblanco, Madrid, España; Servicio de Radiología, Hospital Universitario La Paz – Carlos III – Cantoblanco, Madrid, España

**Keywords:** cateterismo de senos petrosos inferiores, enfermedad de Cushing, estudio de utilidad diagnóstica, síndrome de ACTH ectópico, síndrome de Cushing

## Abstract

**Objetivos:**

El Cateterismo de Senos Petrosos Inferiores (CSSPPII) es una prueba útil para diferenciar entre el origen central y ectópico del síndrome de Cushing hormona adrenocorticotropa (ACTH)-dependiente. Presentamos el protocolo utilizado en nuestro centro y la evaluación de su rendimiento diagnóstico.

**Métodos:**

Estudio retrospectivo de 28 pacientes sometidos a cateterismo de senos petrosos inferiores (CSSPPII) con estímulo por hormona liberadora de corticotropina (CRH). El procedimiento se realiza en un quirófano de neurorradiología en el que participa un equipo multidisciplinar de neurorradiólogos, endocrinólogos y analistas. Se cateterizan ambos senos petrosos y se obtiene una muestra periférica simultánea, en condiciones basales y a los 3,6 y 10 min tras estímulo. Se determinan ACTH y prolactina mediante inmunoquimioluminiscencia.

**Resultados:**

Total de 19 pacientes con enfermedad de Cushing (EC) y 1 paciente con Cushing ectópico (CE) fueron confirmados. En todos los casos el CSSPPII orientó correctamente el diagnóstico, obteniéndose valores de sensibilidad y especificidad del 100%. En 8 pacientes no se alcanzó remisión postquirúrgica de la enfermedad. En el 84% de los cateterismos el valor de ratio más alto se alcanzó entre los 3 y 6 min postestímulo. Ratios y valores de ACTH en seno fueron superiores en los pacientes con confirmación histológica de EC. La ratio de prolactina permitió descartar un 28,6% de las muestras que habrían supuesto resultados discordantes respecto al resto de la exploración.

**Conclusiones:**

En nuestra serie, el CSSPPII con estímulo por CRH ha demostrado ser un procedimiento seguro y eficaz. Se destaca la utilidad de la medición de prolactina como marcador de correcta cateterización y la importancia de la participación de un equipo multidisciplinar.

## Introducción

El síndrome de Cushing (SC) es el conjunto de manifestaciones clínicas resultantes de la exposición prolongada del organismo a una elevada concentración de glucocorticoides. Entre los signos y síntomas más frecuentes se encuentran la ganancia de peso, la hipertensión y la diabetes, siendo importante realizar un diagnóstico diferencial con estas patologías. Otros más característicos son la miopatía, atrofia dérmica, hematomas frecuentes, desórdenes psiquiátricos e hirsutismo [1, 2].

La principal causa de esta patología es la administración exógena de glucocorticoides [1]. Sin embargo, aquellos de etiología endógena representan una entidad con importante relevancia clínica, distinguiéndose los que cursan con niveles elevados de hormona adrenocorticotropa (ACTH) en sangre de los que no [3]. La causa más común del SC ACTH-independiente es la hipersecreción de glucocorticoides por la glándula suprarrenal debido a la presencia de un adenoma o carcinoma. En los ACTH-dependientes podemos diferenciar entre los debidos a una causa central, principalmente como consecuencia de un adenoma hipofisario, y los de causa ectópica por tumores productores de ACTH no hipofisarios. Se denomina enfermedad de Cushing (EC) cuando el hipercortisolismo tiene un origen central, suponiendo un 60–80% del total de casos de Cushing endógeno [1]. Esta entidad presenta una incidencia en Europa de 0,7–2,4 casos por millón de habitantes y año, siendo más prevalente en mujeres que en hombres [4].

Para identificar el origen de ACTH se emplean pruebas de estimulación con CRH o desmopresina y pruebas de supresión con dexametasona a dosis altas [5], junto a las pruebas de imagen, principalmente resonancia magnética (RM). Ambos métodos presentan importantes limitaciones: la inespecificidad de las pruebas de estimulación, el pequeño tamaño de los tumores y la posibilidad de identificar adenomas no funcionantes. El tratamiento de elección en la EC es la resección transesfenoidal de la hipófisis. Por lo tanto, es importante el uso de pruebas con una elevada especificidad para evitar cirugías innecesarias.

En la década de los 70 comenzó a desarrollarse el cateterismo de senos petrosos inferiores (CSSPPII) como forma de localizar el origen de la secreción anómala de ACTH. El primer caso de CSSPPII llevado a cabo en un paciente con SC fue descrito por Corrigan y su grupo de trabajo [6].

El CSSPPII es una prueba diagnóstica en la que participa un equipo multidisciplinar, solicitada e interpretada por el servicio de endocrinología, quienes controlan la retirada de la supresión suprarrenal que suele ser habitual en estos casos, realizada en quirófano por radiólogos intervencionistas y con el posterior procesamiento de las muestras en el laboratorio. Se trata de una prueba invasiva en la que se cateterizan ambos senos petrosos mediante acceso venoso femoral bilateral y, adicionalmente, se canaliza una vía venosa periférica [3, 7]. Se obtienen muestras simultáneamente de las tres localizaciones en situación basal y en diferentes tiempos tras estimulación farmacológica de la hipófisis habitualmente en los 15 min posteriores al estímulo [3, 8], [9], [10]. Para la estimulación se puede utilizar tanto CRH como desmopresina, habiéndose manifestado con su uso un aumento de sensibilidad y especificidad de la prueba [7]. Actualmente, el CSSPPII es la prueba de referencia para diferenciar el origen central o ectópico del SC por el elevado valor diagnóstico que ha demostrado [11].

El fundamento de esta prueba es que en el caso de que exista EC la concentración de ACTH será significativamente mayor en las muestras procedentes de los senos que en las muestras periféricas [12]. Para facilitar la interpretación de esta diferencia de concentraciones, se implementó el uso de ratios de ACTH seno/periferia. Fueron Oldfield et al. quienes definieron que una ratio ≥2 en muestras basales o una ratio ≥3 tras estímulo era indicativo de EC, mientras que si eran menores se sospecharía una causa ectópica [13]. En la actualidad se siguen utilizando estos puntos de corte.

En el intento de minimizar los falsos negativos (pacientes con EC y resultado no sugestivo de adenoma productor de ACTH en el cateterismo), que puedan deberse a una incorrecta cateterización o a variaciones en la circulación venosa que alteran el drenaje habitual de la hipófisis, se ha propuesto la utilización de los niveles de prolactina como indicador de cateterización correcta [11], utilizando el índice de selectividad (IS), definido como la ratio de prolactina seno/periferia [14].

El CCSSPPII se emplea en pacientes con SC en los que previamente se ha demostrado bioquímicamente que la hipercortisolemia es dependiente de la producción de ACTH, con el objetivo de confirmar el origen central o ectópico. Su uso está especialmente indicado si las pruebas funcionales no invasivas son discordantes entre ellas, o cuando no se observa adenoma en la prueba de imagen o presenta un tamaño inferior a 6 mm [2, 3, 15, 16]. Esta intervención conlleva ciertos riesgos para el paciente, como malestar general o hematoma inguinal postcateterismo, que pueden verse en el 1–4% de los pacientes, y con mucha menor incidencia eventos trombóticos [1, 3].

También se ha sugerido la utilidad del CSSPPII para obtener información sobre la localización del adenoma en la hipófisis y orientar la exploración quirúrgica. El estudio de la lateralización se realiza calculándose el gradiente de ACTH entre senos (ratio ACTH seno derecho/seno izquierdo, o viceversa), considerándose predictivo de lateralización cuando la ratio es ≥1,4 [9, 12].

En nuestro hospital se estandarizó el uso del CCSSPPII con estimulación mediante CRH a partir del año 2001. A partir del año 2014 se añadió la determinación de prolactina. Presentamos en este estudio la evaluación de la serie de pacientes atendidos en este período y el rendimiento diagnóstico obtenido.

## Materiales y métodos

Se ha realizado un estudio retrospectivo de los pacientes sometidos a CSSPPII en el Hospital Universitario La Paz entre los años 2001 y 2020.

El procedimiento de cateterización de ambos senos petrosos se realiza mediante acceso venoso femoral bilateral y, adicionalmente, se canaliza una vía venosa periférica. El proceso es llevado a cabo por 2 radiólogos intervencionistas experimentados en un quirófano de neurorradiología del Servicio de Radiología.

Se recogen muestras de cada localización de forma simultánea, en condiciones basales y a los 3, 6 y 10 min tras la administración en bolo de 100 μg de CRH intravenosa. Este protocolo ha variado en cuanto a los tiempos utilizados. Inicialmente, hasta el año 2011 se extraían muestras en el momento basal y a los 6 y 10 min postestímulo, incorporándose desde entonces la recogida en el minuto 3.

Para evitar el riesgo de contaminación por el contraste utilizado durante la prueba, debe disminuirse su uso al máximo y purgar adecuadamente antes de la obtención de la muestra. En nuestro caso la muestra se obtiene utilizando dos jeringas de 5 mL. La primera se utiliza como purga y, a continuación, se obtienen entre 2 y 4 mL de sangre.

Para asegurar que no hay posibilidad de confusión en las muestras obtenidas, el personal de laboratorio participa en la obtención en quirófano, encargándose de alicuotar las muestras de cada localización de acuerdo con el instrumentista. De cada tiempo y localización se reparte la muestra obtenida en un tubo de suero con gel separador para la medición de prolactina, y un tubo con anticoagulante EDTA 2K con gel separador para la medición de ACTH. Para evitar la degradación tiempo y temperatura dependiente de la ACTH [17], las muestras se mantienen en frío durante el transporte hasta el laboratorio, donde se centrifugan y procesan en el mínimo tiempo posible. La medición de ACTH se realiza en el analizador Immulite (Siemens Healthineers) y la prolactina en Advia Centaur (Siemens Healthineers) hasta el año 2018, que comienza a utilizarse Atellica Solution (Siemens Healthineers). En todos ellos se utiliza inmunoquimioluminiscencia como método de detección.

Con los datos de ACTH obtenidos, se calculan las ratios ACTH seno/periferia. Se considera posible EC cuando la ratio es ≥2 en el momento basal o ≥3 en las muestras postestímulo. Una ratio superior en alguno de los senos y en alguno de los cuatro tiempos es suficiente para confirmar origen central [3].

Con los cambios realizados en el protocolo en el año 2011, se incorporó la medición de prolactina como indicador de correcta cateterización. A partir de entonces, se comienza a utilizar el IS como requisito previo para considerar valorable la ratio ACTH seno/periferia. De cada una de las muestras de los senos, solo se consideran informativas aquellas cuyo IS fuera ≥1,8.

El diagnóstico de EC fue confirmado mediante el hallazgo histológico de adenoma secretor de ACTH y/o curación tras la cirugía.

Respecto a la lateralización, estudiamos la eficacia del CSSPPII y de la RM, tomando como resultado de referencia la visualización del adenoma durante la cirugía o en el estudio anatomopatológico.

Todos los datos presentados corresponden a la asistencia clínica habitual de los pacientes, siguiendo las normas éticas y de conducta aprobadas en el protocolo de Helsinki.

Se utilizó el programa de análisis estadístico R (versión 4.0.2) tanto para la elaboración de figuras representativas de los datos como para la comparación entre grupos de pacientes, aplicando el test de Kruskal–Wallis.

## Resultados

Durante el período estudiado se sometieron a CSSPPII 28 pacientes, con una edad media de 43,36 ± 11,89 años, 24 mujeres (85,7%) y 4 hombres (14,3%). Ninguno desarrolló complicaciones derivadas de la intervención. En solo uno de los pacientes se tuvo que repetir el procedimiento por dificultades en la obtención de muestras.

De los 28 pacientes, en 26 se obtuvieron resultados de ratio ACTH seno/periferia superiores al punto de corte, compatibles con un origen central. Todos ellos fueron sometidos a cirugía transesfenoidal. La EC fue confirmada en 19 casos, 11 por hallazgo de adenoma secretor de ACTH y 8 por cumplir criterios de curación postcirugía. En los 7 pacientes restantes no se ha podido verificar el origen central (Tabla 1).

**Tabla 1:** Distribución de pacientes según resultado de ratio ACTH y resultado postcirugía.

Resultados CSSPPII n total=28
Ratio ACTH seno/periferia ≥2 basal y/o ≥3 postestímulo=26
– EC confirmada 19
– EC sin confirmar 7
Ratio ACTH seno/periferia <2 basal y<3 postestímulo=2
– CE confirmado 1
– CE sin confirmar 1

Los 2 pacientes restantes presentaron valores de ratio ACTH seno/periferia inferiores al punto de corte, sugestivos de origen ectópico. En uno se confirmó por inmunohistoquímica la presencia de un tumor carcinoide de pulmón secretor de ACTH. El otro paciente se sometió a lobectomía pulmonar por la presencia de una imagen sospechosa, pero no pudo confirmarse la presencia de tumor en la pieza quirúrgica, manteniéndose sintomático tras la intervención. En este caso no se pudo descartar una incorrecta cateterización, por haberse realizado en el período en el que aún no se utilizaba el IS.

Considerando los 20 pacientes con diagnóstico confirmado, los valores de sensibilidad y especificidad que nos aporta el CSSPPII para el diagnóstico de EC o CE son del 100%. Estos valores se alcanzan tanto en el momento basal como en los tiempos postestímulos.

**Figura 1:**
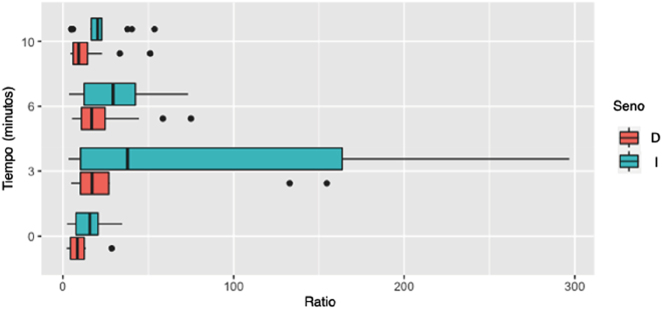
Representación de las ratios de ACTH seno/periferia a los diferentes tiempos de los pacientes con EC confirmada.

Para identificar el tiempo en el que se consiguen valores de ratios más elevados, se evaluaron las ratios alcanzadas a los diferentes tiempos en aquellos pacientes con EC confirmada. En 16 cateterismos (84%) la ratio más alta se alcanzó entre los tiempos 3 y 6 min postestímulo, en 1 (5%) a tiempo basal y en 2 (11%) a tiempo 10 tras estímulo. Al representar las ratios indicativas de EC, se observa que en el tiempo 3 es en el que se alcanzan mayores ratios (Figura 1).

**Figura 2:**
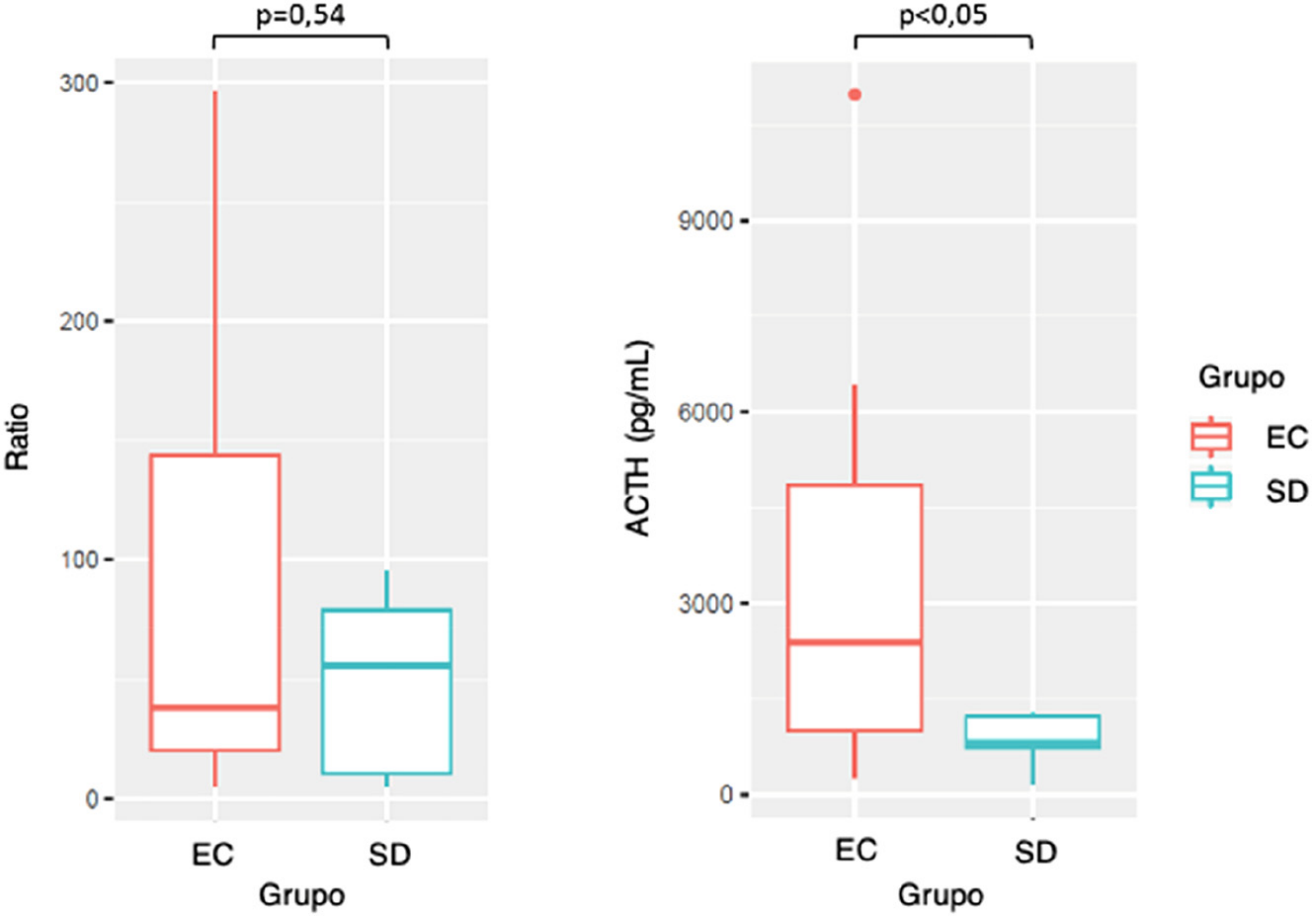
Diferencias en cuanto a valores de ratio y niveles ACTH máximos en los senos entre el grupo de pacientes con EC y el grupo de pacientes SD.

Considerando todos los pacientes con resultados de CSSPPII compatibles con un origen central, se analizaron las posibles diferencias entre el grupo con EC confirmada y aquellos sin diagnóstico definitivo (SD), en cuanto a valores de ratio y niveles de ACTH en los senos. No se observaron diferencias estadísticamente significativas entre los valores de ratio de ambos grupos. Sin embargo, los niveles de ACTH fueron significativamente mayores en el grupo de pacientes con EC (Figura 2).

De todos los CCSSPPII, en 21 de ellos se ha utilizado el IS de prolactina como indicador de correcta cateterización. En 7 cateterismos se obtuvo un IS ≥1,8 en solo uno de los senos y en el resto se consiguió una correcta cateterización de ambos senos en al menos uno de los tiempos. Considerando todos los tiempos de los 21 pacientes, la muestra era representativa de drenaje hipofisario en el seno derecho en el 76,2% (64/84) de las muestras y en el izquierdo en el 66,7% (56/84). En conjunto, la localización del catéter era adecuada en el momento de recogida de la muestra en un 71,4% (120/168) de los casos.

En los 13 pacientes con EC confirmada en los que se calculó el IS, de las 104 muestras obtenidas, en 25 el IS era inferior al punto de corte sugiriendo que la muestra no era representativa. En la mayoría de estos puntos no informativos (16 puntos pertenecientes a 6 pacientes), la ratio ACTH era inferior al punto de corte sugiriendo origen ectópico, discordante con el resto de la exploración.

Se estudiaron los resultados de lateralización derivados del CCSSPPII considerando los 17 casos cuya cirugía aportó un resultado concluyente, con confirmación de Anatomía Patológica de la localización intrahipofisaria del tumor. Para interpretar la lateralización se aplicó el criterio de ratio ACTH ≥1,4 entre senos. En 2 pacientes se observaron discrepancias en la lateralización en los 4 tiempos de la prueba, por lo que se clasificaron como indeterminados. Se obtuvo una concordancia del 64,7% respecto a anatomía patológica (Tabla 2), inferior a los resultados derivados del estudio de imagen mediante RM, disponible en 14 casos, en los que se observa una concordancia del 85,7%.

**Tabla 2:** Concordancia entre los resultados de lateralización derivados del CSSPPII y los obtenidos por Anatomía Patológica tras cirugía.

		Lateralización según resultado CSSPII (ratio ACTH entre senos ≥1,4)		
		Derecha	Indeterminada	Izquierda
Lateralización observada en Anatomía Patológica	Derecha (9)	5 (56%)	2 (22%)	2 (22%)
	Izquierda (8)	2 (25%)	0	6 (75%)

## Discusión

El diagnóstico de la EC puede suponer un reto. Las pruebas no invasivas son la herramienta inicial para comprobar si existe dependencia o no de la producción de ACTH y para diferenciar el origen central o ectópico de esta. Sin embargo, sus resultados no siempre son suficientes para aconsejar el abordaje quirúrgico. Es en estos pacientes en los que el CSSPPII cobra una mayor relevancia [18, 19]. En nuestro estudio, el resultado de la prueban de imagen de los 28 pacientes sometidos a CSSPPII fue negativo en 7 de ellos. De los 19 restantes, el tamaño del adenoma fue de 10 mm en 1 caso y ≤9 mm en el resto, compatible con la recomendación de la reciente guía sobre el diagnóstico y manejo de la EC [11].

En nuestra serie, el CCSSPPII fue clave para diagnosticar un caso de CE que no había podido ser detectado por otras pruebas. Las ratios seno/periferia de ACTH más elevadas fueron 1,36 y 1,97 en la muestra basal y postestímulo, respectivamente (correspondientes a valores de ACTH en seno de 139 y 187 pg/mL). Es relevante mencionar que el IS de prolactina mostró valores de correcta cateterización en ambos senos en todos los tiempos.

En otro paciente con ratios de ACTH sugestivas de CE la investigación posterior sugirió una posible neoplasia pulmonar, pero no se pudo confirmar histológicamente y tampoco se obtuvo una curación completa posterior a la lobectomía. En este momento no utilizábamos todavía la medición de prolactina para confirmar el origen hipofisario del drenaje venoso.

En los 19 casos de EC confirmada y curada, la ratio seno/periferia más informativa durante el procedimiento oscilaba entre 5,8 y 296,8, que se corresponde a concentraciones de ACTH en seno entre 327 y 10,980 pg/mL, respectivamente. En el 84% de los cateterismos realizados la ratio máxima se alcanzó entre los tiempos 3 y 6 min tras estímulo.

Wind et al. [9] observan, en una serie de 470 casos de EC diagnosticada histológicamente sometidos a CCSSPPII, que las ratios de ACTH se correlacionan muy bien con la concentración de ACTH en los senos y que valores de ACTH <200 pg/mL en la muestra basal y<400 pg/mL tras CRH se relacionan con falsos negativos para EC.

En nuestra serie, al comparar los niveles de ACTH en el seno petroso y las ratios obtenidas entre los 19 pacientes con EC confirmada y los 7 pacientes en los que no se pudo confirmar el origen central, no existen diferencias significativas entre las ratios obtenidas de los 2 grupos, pero sí hay significación estadística en los niveles de ACTH que se obtienen en uno y otro grupo, obteniendo valores de ACTH significativamente mayores en el grupo de EC confirmada.

En 7 casos con resultado de cateterismo sugerente de origen central no se logró la remisión completa de los síntomas tras la cirugía de hipófisis. En el seguimiento de estos pacientes no se ha considerado indicado un nuevo abordaje quirúrgico, por lo que no se ha podido confirmar el origen central de estos casos, que sigue siendo la principal sospecha.

Teniendo en cuenta sólo los casos con curación confirmada, la eficacia diagnóstica del CCSSPPII en nuestra serie es del 100%, criterio similar al utilizado en otras series que muestran valores de sensibilidad entre 85–100% y de especificidad entre 67–100% [3, 12]. Sin embargo, los casos sin confirmación histológica o criterio de curación han sido el 29% en nuestra serie. Esta cifra es similar a la presentada en otros estudios [19]. La remisión postquirúrgica del SC no siempre se consigue, esto sucede tanto en las series que no utilizan el CSSPPII como en las que sí.

La obtención de una muestra basal y 3 muestras postestímulo dificulta el procedimiento, ya que requiere que el catéter esté correctamente posicionado durante más tiempo, pero permite asegurar la obtención de alguna muestra informativa. Durante el procedimiento se debe limitar el uso de contraste para determinar la morfología del sistema de drenaje venoso y el punto de colocación adecuado del catéter, ya que aumenta la necesidad de purgado posterior para la obtención de las muestras y la irradiación del paciente. La manipulación de las sondas durante la obtención de las muestras o la necesidad de movilizar al paciente pueden provocar el desplazamiento del catéter, diluyendo la sangre procedente de la hipófisis con el resto de drenaje cerebral. Por este motivo, la mayoría de las series publicadas recomiendan la toma de varias muestras, habitualmente entre los 3 y 15 min. En nuestro estudio hemos observado que el pico de secreción de ACTH se sitúa habitualmente entre los 3–6 min del estímulo.

El CCSSPPII es especialmente útil en los casos en los que no se observa presencia de adenoma hipofisario en las pruebas de imagen. En esta situación, los resultados falsamente negativos, alrededor del 10% en la mayoría de los estudios [7], se han relacionado con la obtención de muestras que no son representativas. Debe tenerse en cuenta la existencia de variantes anatómicas en el sistema vascular de los senos petrosos, que conlleva un drenaje anómalo de la hipófisis y genera una mayor dificultad para la colocación del catéter [20, 21].

Se han descrito 6 tipos de variantes en la circulación venosa cerebral, dependiendo de cómo se produzca el drenaje de sangre procedente de los senos petrosos inferiores en la vena yugular [21]. Estas variantes se pueden dar de forma bilateral o unilateral y pueden dificultar el acceso correcto, así como provocar un drenaje desigual de los hemisferios hipofisarios, pudiendo dar lugar a confusión en el IS y en el diagnóstico de lateralización.

Una forma a posteriori de evaluar la calidad de la muestra hipofisaria es utilizar la medición simultánea de prolactina, ya que se trata de una hormona de secreción hipofisaria en zonas habitualmente separadas de las corticotropas y con mayor vida media en la circulación. Un IS ≥1,8 permite seleccionar las muestras con mayor probabilidad de recogida de sangre drenada por la hipófisis, aumentando la especificidad del CSSPPII [22].

Algunos autores [14, 22] recomiendan además la normalización de las ratios ACTH respecto a las ratios de prolactina, de tal forma que una ratio normalizada >0,8 sería indicativa de EC, a pesar de considerar que no se ha conseguido un efluente hipofisario adecuado. Otras publicaciones [23, 24] sugieren que esta normalización podría ser errónea en pacientes con CE y recomiendan exclusivamente el uso de la ratio de prolactina para identificar los puntos informativos de la prueba.

En nuestra serie, a partir de la utilización sistemática del IS, hemos objetivado un 28,6% de las tomas con sospecha de localización inadecuada del catéter, que habitualmente suponían resultados discordantes en la exploración. En 6 pacientes con EC confirmada uno de los senos podría haberse interpretado como negativo si no se hubiera utilizado el IS. En el caso del paciente con CE confirmado, el IS apoyaba la correcta cateterización y daba más fuerza a la necesidad de exploraciones adicionales en busca del origen ectópico.

La confirmación pre-quirúrgica de la lateralización puede ser una herramienta útil para llevar a cabo un abordaje más conservador, evitando la cirugía hipofisaria completa. En nuestra serie encontramos que tanto la lateralización bioquímica como la observada en las pruebas de imagen se correlacionan bastante bien con la localización quirúrgica (64,7% y 85,7% respectivamente). Los resultados de lateralización bioquímica obtenidos en nuestra serie son similares a los descritos en bibliografía [2, 9, 21].

El CCSSPPII se ha consolidado en múltiples series de casos como método de referencia para diferenciar entre EC y CE [3, 8, 10, 12, 15]. Se trata de una prueba invasiva que precisa de unas instalaciones adecuadas y un equipo multidisciplinar especializado, pero con alto porcentaje de éxito y escasas complicaciones. La colaboración del laboratorio durante la realización del CSSPPII es fundamental para asegurar un correcto procesamiento de unas muestras extremadamente valiosas. La obtención de múltiples tiempos tras estímulo y la determinación de prolactina son recomendables para mejorar la eficacia diagnóstica del test.
